# Supplementary material for: A New Application of Social Impact in Social Media for Overcoming Fake News in Health
Source: Int J Environ Res Public Health. 2020 Apr 3;17(7):2430. doi: 10.3390/ijerph17072430 (PMC7177765; doi:10.3390/ijerph17072430)
Supplement: Supplementary file 1 [file ijerph-17-02430-s001.pdf]

Cases	Rater A	Rater B
Twitter_1	1	1
Twitter_2	1	1
Twitter_3	1	1
Twitter_4	1	1
Twitter_5	1	1
Twitter_6	1	1
Twitter_7	1	1
Twitter_8	1	1
Twitter_9	1	1
Twitter_10	1	1
Twitter_11	1	1
Twitter_12	1	1
Twitter_13	1	1
Twitter_14	1	1
Twitter_15	1	1
Twitter_16	1	1
Twitter_17	1	1
Twitter_18	1	1
Twitter_19	1	1
Twitter_20	1	1
Twitter_21	1	1
Twitter_22	1	1
Twitter_23	1	1
Twitter_24	1	1
Twitter_25	1	1
Twitter_26	1	1
Twitter_27	1	1
Twitter_28	1	1
Twitter_29	1	1
Twitter_30	1	1
Twitter_31	1	1
Twitter_32	1	1
Twitter_33	1	1
Twitter_34	1	1
Twitter_35	1	1
Twitter_36	1	1
Twitter_37	1	1
Twitter_38	1	1
Twitter_39	2	2
Twitter_40	2	2
Twitter_41	2	2
Twitter_42	2	1
Twitter_43	2	2
Twitter_44	2	2
Twitter_45	2	2
Twitter_46	2	2
Twitter_47	2	2
Twitter_48	2	2
Twitter_49	2	2
Twitter_50	2	2
Twitter_51	2	2
Twitter_52	2	2

Cases where Raters do not coincide

Twitter_42	2	1	Agree to exclude
Twitter_54	2	1	Agree to exclude
Twitter_59	2	1	Rater A want to include

ESISM	1
INFO	2
MISFA	3
OPINION	4
Sample	453

Both raters are agree to include	450	
Both raters are agree to exclude	2	450
**Rater A want to include**	1	
**Rater B want to exclude**	0	

Cohen’s k 0.79 Substantial agreement

Total number	453
Excluded	3
**Final sample**	**450**

*** Those cases that are not evaluated with the same value are excluded of the final analysis

Cohen’s kappa calculator
Available in
https://idostatistics.com/cohen-kappa-free-calculator/#risultati

Twitter_53	2	2
Twitter_54	2	1
Twitter_55	2	2
Twitter_56	2	2
Twitter_57	2	2
Twitter_58	2	2
Twitter_59	2	1
Twitter_60	2	2
Twitter_61	2	2
Twitter_62	2	2
Twitter_63	2	2
Twitter_64	2	2
Twitter_65	2	2
Twitter_66	2	2
Twitter_67	2	2
Twitter_68	2	2
Twitter_69	2	2
Twitter_70	2	2
Twitter_71	2	2
Twitter_72	2	2
Twitter_73	2	2
Twitter_74	2	2
Twitter_75	2	2
Twitter_76	2	2
Twitter_77	2	2
Twitter_78	2	2
Twitter_79	2	2
Twitter_80	2	2
Twitter_81	2	2
Twitter_82	2	2
Twitter_83	2	2
Twitter_84	2	2
Twitter_85	2	2
Twitter_86	2	2
Twitter_87	2	2
Twitter_88	2	2
Twitter_89	2	2
Twitter_90	2	2
Twitter_91	3	3
Twitter_92	3	3
Twitter_93	3	3
Twitter_94	4	4
Twitter_95	4	4
Twitter_96	4	4
Twitter_97	4	4
Twitter_98	4	4
Twitter_99	4	4
Twitter_100	4	4
Twitter_101	1	1
Twitter_102	1	1
Twitter_103	1	1
Twitter_104	1	1
Twitter_105	1	1
Twitter_106	1	1
Twitter_107	1	1

Twitter_108	1	1
Twitter_109	1	1
Twitter_110	1	1
Twitter_111	1	1
Twitter_112	1	1
Twitter_113	1	1
Twitter_114	1	1
Twitter_115	1	1
Twitter_116	1	1
Twitter_117	1	1
Twitter_118	1	1
Twitter_119	2	2
Twitter_120	2	2
Twitter_121	2	2
Twitter_122	2	2
Twitter_123	2	2
Twitter_124	2	2
Twitter_125	2	2
Twitter_126	2	2
Twitter_127	2	2
Twitter_128	2	2
Twitter_129	2	2
Twitter_130	2	2
Twitter_131	2	2
Twitter_132	2	2
Twitter_133	2	2
Twitter_134	2	2
Twitter_135	2	2
Twitter_136	2	2
Twitter_137	2	2
Twitter_138	2	2
Twitter_139	2	2
Twitter_140	2	2
Twitter_141	2	2
Twitter_142	2	2
Twitter_143	2	2
Twitter_144	2	2
Twitter_145	2	2
Twitter_146	2	2
Twitter_147	2	2
Twitter_148	2	2
Twitter_149	2	2
Twitter_150	2	2
Twitter_151	2	2
Twitter_152	2	2
Twitter_153	2	2
Twitter_154	2	2
Twitter_155	2	2
Twitter_156	2	2
Twitter_157	2	2
Twitter_158	2	2
Twitter_159	2	2
Twitter_160	2	2
Twitter_161	2	2
Twitter_162	2	2

Twitter_163	2	2
Twitter_164	2	2
Twitter_165	2	2
Twitter_166	2	2
Twitter_167	2	2
Twitter_168	2	2
Twitter_169	2	2
Twitter_170	3	3
Twitter_171	3	3
Twitter_172	3	3
Twitter_173	3	3
Twitter_174	3	3
Twitter_175	3	3
Twitter_176	3	3
Twitter_177	3	3
Twitter_178	3	3
Twitter_179	3	3
Twitter_180	3	3
Twitter_181	3	3
Twitter_182	3	3
Twitter_183	3	3
Twitter_184	3	3
Twitter_185	3	3
Twitter_186	3	3
Twitter_187	3	3
Twitter_188	3	3
Twitter_189	3	3
Twitter_190	3	3
Twitter_191	4	4
Twitter_192	4	4
Twitter_193	4	4
Twitter_194	4	4
Twitter_195	4	4
Twitter_196	4	4
Twitter_197	4	4
Twitter_198	4	4
Twitter_199	4	4
Twitter_200	4	4
Twitter_201	1	1
Twitter_202	1	1
Twitter_203	1	1
Twitter_204	1	1
Twitter_205	2	2
Twitter_206	2	2
Twitter_207	2	2
Twitter_208	2	2
Twitter_209	2	2
Twitter_210	2	2
Twitter_211	2	2
Twitter_212	2	2
Twitter_213	2	2
Twitter_214	2	2
Twitter_215	2	2
Twitter_216	2	2
Twitter_217	2	2

Twitter_218	2	2
Twitter_219	2	2
Twitter_220	2	2
Twitter_221	2	2
Twitter_222	2	2
Twitter_223	2	2
Twitter_224	2	2
Twitter_225	2	2
Twitter_226	2	2
Twitter_227	2	2
Twitter_228	2	2
Twitter_229	2	2
Twitter_230	2	2
Twitter_231	2	2
Twitter_232	2	2
Twitter_233	2	2
Twitter_234	2	2
Twitter_235	2	2
Twitter_236	2	2
Twitter_237	2	2
Twitter_238	2	2
Twitter_239	2	2
Twitter_240	3	3
Twitter_241	3	3
Twitter_242	3	3
Twitter_243	3	3
Twitter_244	3	3
Twitter_245	3	3
Twitter_246	3	3
Twitter_247	3	3
Twitter_248	3	3
Twitter_249	3	3
Twitter_250	3	3
Twitter_251	3	3
Twitter_252	3	3
Twitter_253	3	3
Twitter_254	3	3
Twitter_255	3	3
Twitter_256	3	3
Twitter_257	3	3
Twitter_258	3	3
Twitter_259	3	3
Twitter_260	3	3
Twitter_261	3	3
Twitter_262	3	3
Twitter_263	3	3
Twitter_264	3	3
Twitter_265	3	3
Twitter_266	3	3
Twitter_267	3	3
Twitter_268	3	3
Twitter_269	3	3
Twitter_270	3	3
Twitter_271	3	3
Twitter_272	4	4

Twitter_273	4	4
Twitter_274	4	4
Twitter_275	4	4
Twitter_276	4	4
Twitter_277	4	4
Twitter_278	4	4
Twitter_279	4	4
Twitter_280	4	4
Twitter_281	4	4
Twitter_282	4	4
Twitter_283	4	4
Twitter_284	4	4
Twitter_285	4	4
Twitter_286	4	4
Twitter_287	4	4
Twitter_288	4	4
Twitter_289	4	4
Twitter_290	4	4
Twitter_291	4	4
Twitter_292	4	4
Twitter_293	4	4
Twitter_294	4	4
Twitter_295	4	4
Twitter_296	4	4
Twitter_297	4	4
Twitter_298	4	4
Twitter_299	4	4
Twitter_300	4	4
Facebook_1	1	1
Facebook_2	1	1
Facebook_3	1	1
Facebook_4	1	1
Facebook_5	1	1
Facebook_6	1	1
Facebook_7	2	2
Facebook_8	2	2
Facebook_9	2	2
Facebook_10	2	2
Facebook_11	2	2
Facebook_12	2	2
Facebook_13	2	2
Facebook_14	2	2
Facebook_15	2	2
Facebook_16	2	2
Facebook_17	2	2
Facebook_18	2	2
Facebook_19	2	2
Facebook_20	2	2
Facebook_21	2	2
Facebook_22	2	2
Facebook_23	2	2
Facebook_24	2	2
Facebook_25	2	2
Facebook_26	3	3
Facebook_27	3	3

Facebook_28	4	4
Facebook_29	4	4
Facebook_30	4	4
Facebook_31	4	4
Facebook_32	4	4
Facebook_33	4	4
Facebook_34	4	4
Facebook_35	4	4
Facebook_36	4	4
Facebook_37	4	4
Facebook_38	4	4
Facebook_39	4	4
Facebook_40	4	4
Facebook_41	2	2
Facebook_42	2	2
Facebook_43	2	2
Facebook_44	2	2
Facebook_45	2	2
Facebook_46	2	2
Facebook_47	2	2
Facebook_48	2	2
Facebook_49	2	2
Facebook_50	2	2
Facebook_51	2	2
Facebook_52	2	2
Facebook_53	2	2
Facebook_54	2	2
Facebook_55	2	2
Facebook_56	2	2
Facebook_57	2	2
Facebook_58	3	3
Facebook_59	4	4
Facebook_60	4	4
Facebook_61	4	4
Facebook_62	4	4
Facebook_63	4	4
Facebook_64	4	4
Facebook_65	4	4
Facebook_66	4	4
Facebook_67	4	4
Facebook_68	4	4
Facebook_69	4	4
Facebook_70	4	4
Facebook_71	4	4
Facebook_72	4	4
Facebook_73	4	4
Facebook_74	4	4
Facebook_75	4	4
Facebook_76	4	4
Facebook_77	4	4
Facebook_78	4	4
Facebook_79	4	4
Facebook_80	4	4
Reddit_1	1	1
Reddit_2	1	1

Reddit_3	1	1
Reddit_4	1	1
Reddit_5	1	1
Reddit_6	1	1
Reddit_7	1	1
Reddit_8	1	1
Reddit_9	1	1
Reddit_10	1	1
Reddit_11	1	1
Reddit_12	1	1
Reddit_13	1	1
Reddit_14	1	1
Reddit_15	2	2
Reddit_16	2	2
Reddit_17	2	2
Reddit_18	2	2
Reddit_19	2	2
Reddit_20	2	2
Reddit_21	2	2
Reddit_22	2	2
Reddit_23	2	2
Reddit_24	2	2
Reddit_25	2	2
Reddit_26	2	2
Reddit_27	2	2
Reddit_28	2	2
Reddit_29	2	2
Reddit_30	2	2
Reddit_31	2	2
Reddit_32	2	2
Reddit_33	2	2
Reddit_34	3	3
Reddit_35	3	3
Reddit_36	3	3
Reddit_37	4	4
Reddit_38	4	4
Reddit_39	4	4
Reddit_40	4	4
Reddit_41	4	4
Reddit_42	4	4
Reddit_43	4	4
Reddit_44	4	4
Reddit_45	4	4
Reddit_46	4	4
Reddit_47	4	4
Reddit_48	4	4
Reddit_49	4	4
Reddit_50	4	4
Reddit_51	4	4
Reddit_52	4	4
RedditASK_1	1	1
RedditASK_2	1	1
RedditASK_3	1	1
RedditASK_4	2	2
RedditASK_5	2	2

RedditASK_6	2	2
RedditASK_7	2	2
RedditASK_8	2	2
RedditASK_9	2	2
RedditASK_10	2	2
RedditASK_11	2	2
RedditASK_12	2	2
RedditASK_13	2	2
RedditASK_14	2	2
RedditASK_15	2	2
RedditASK_16	2	2
RedditASK_17	3	3
RedditASK_18	3	3
RedditASK_19	4	4
RedditASK_20	4	4
RedditASK_21	4	4
